# The *MUC5B* Promoter Polymorphism Associates With Severe COVID-19 in the European Population

**DOI:** 10.3389/fmed.2021.668024

**Published:** 2021-11-23

**Authors:** Coline H. M. van Moorsel, Joanne J. van der Vis, Anna Duckworth, Chris J. Scotton, Claudia Benschop, David Ellinghaus, Henk J. T. Ruven, Marian J. R. Quanjel, Jan C. Grutters

**Affiliations:** ^1^Department of Pulmonology, St Antonius ILD Center of Excellence, St. Antonius Hospital, Nieuwegein, Netherlands; ^2^Division of Heart and Lungs, University Medical Center Utrecht, Utrecht, Netherlands; ^3^Department of Clinical Chemistry, St Antonius ILD Center of Excellence, St. Antonius Hospital, Nieuwegein, Netherlands; ^4^College of Medicine & Health, Institute of Biomedical & Clinical Science, University of Exeter, Exeter, United Kingdom; ^5^Department of Medical Microbiology and Immunology, St Antonius ILD Center of Excellence, St. Antonius Hospital, Nieuwegein, Netherlands; ^6^Genetics and Bioinformatics Group, Institute of Clinical Molecular Biology, Christian-Albrechts-University, Kiel, Germany; ^7^Faculty of Health and Medical Sciences, Novo Nordisk Foundation Center for Protein Research, Disease Systems Biology, University of Copenhagen, Copenhagen, Denmark

**Keywords:** *MUC5B*, COVID-19, idiopathic pulmonary fibrosis, innate immunity, mucus, SARS-CoV-2, aging lung

## Abstract

**Background:** Diversity in response on exposure to severe acute respiratory syndrome coronavirus 2 may be related to the innate immune response in the elderly. The mucin MUC5B is an important component of the innate immune response and expression levels are associated with the *MUC5B* promoter polymorphism, rs35705950. The high expressing T-allele is a risk allele for the non-infectious aging lung disease idiopathic pulmonary fibrosis (IPF). We investigated if *MUC5B* rs35705950 associates with severe COVID-19.

**Methods:** In this retrospective candidate gene case-control study we recruited 108 Dutch patients (69% male, median age 66 years, 77% white) requiring hospitalization for COVID-19 (22% ICU stay, 24% died). For validation, genotypes were obtained from the UK-Biobank (*n* = 436, 57% male, median age 70 years, 27% died), for replication data from the severe COVID-19 GWAS group from Italy (*n* = 835) and Spain (*n* = 775) was used, each with a control cohort (*n* = 356,735, *n* = 1,255, *n* = 950, respectively). *MUC5B* association analysis was performed including adjustment for age and sex.

**Results:** The rs35705950 T-allele frequency was significantly lower in Dutch white patients (*n* = 83) than in controls (0.04 vs. 0.10; *p* = 0.02). This was validated in the UK biobank cohort (0.08 vs. 0.11; *p* = 0.001). While age and sex differed significantly between cases and control, comparable results were obtained with age and sex as confounding variables in a multivariate analysis. The association was replicated in the Italian (*p* = 0.04), and Spanish (*p* = 0.03) case-control cohorts. Meta-analysis showed a negative association for the T-allele with COVID-19 (OR = 0.75 (CI: 0.67–0.85); *p* = 6.63 × 10^−6^).

**Conclusions:** This study shows that carriage of the T-allele of *MUC5B* rs35705950 confers protection from development of severe COVID-19. Because the T-allele is a known risk allele for IPF, this study provides further evidence for the existence of trade-offs between optimal mucin expression levels in the aging lung.

## Introduction

The current coronavirus disease (COVID-19) pandemic illustrates the diversity in response on exposure to severe acute respiratory syndrome coronavirus 2 (SARS-CoV-2). Response to infection ranges from asymptomatic to death from organ failure, of which the latter is most commonly observed in the elderly (1). Such differences are associated with aging, but may also be influenced by the genetic constitution of the host.

Diversity in response to SARS-CoV-2 exposure may be related to host factors associated with airway defense. The gel-forming mucin 5B (MUC5B) is part of the mucus that covers the surface of the respiratory epithelium and forms a key barrier defense against respiratory pathogens (2, 3). *In vivo* studies in mice showed that Muc5B deficiency caused accumulation of materials in the upper and lower airways, leading to chronic infection and inflammation that failed to resolve normally. By contrast, in mice that overexpress Muc5B, macrophage function was improved. Hence, the presence of Muc5B in the lung is essential for controlling infections, maintaining immune homeostasis, and mucociliary clearance (4). Aged mice had significantly reduced Muc5b levels in comparison with young mice (5) and decreased expression of Muc5B in mouse models was associated with reduced mucociliary clearance (4, 5). In both humans (6) and mice (5) decreased mucociliary clearance was shown to be associated with aging.

Constitutive expression levels of MUC5B are associated with a common promoter polymorphism, rs35705950 of the encoding gene *MUC5B*. The minor rs35705950 T allele is associated with high expression levels of MUC5B and the major G allele is associated with low expression levels (7, 8). The high expressing T-allele is a known risk factor for idiopathic pulmonary fibrosis (IPF) (7), a fatal aging lung disease of unknown cause predominately affecting older males with a history of smoking. IPF is a non-infectious disease of the distal lung caused by damage of the alveolar epithelium followed by progressive fibrogenesis (9).

Recently it was shown that aging lung diseases such as IPF and chronic obstructive pulmonary disease (COPD) share disease loci but have opposite risk alleles (10). Given the fact that the alleles of these loci influence expression levels we proposed a theory of trade-offs in aging lung disease (11). A trade-off exists whenever a benefit in one context entails a cost in another (12). In aging lungs, the high expressing *MUC5B* T-allele may be important for optimal airway defense against infections while it provides an increased risk for IPF in the alveolar compartment.

Therefore, we examined if *MUC5B* rs35705950 is associated with severe COVID-19. To investigate this, we performed a retrospective candidate rs35705950 case-control study in a Dutch cohort and included an UK cohort for validation and an Italian and Spanish cohort for replication.

## Materials and Methods

### Patients

This is a retrospective candidate gene case-control study. The discovery cohort from the ILD biobank and data registry of the St Antonius Hospital Nieuwegein, the Netherlands, included (*n* = 108) adult patients hospitalized due to COVID-19 at St Antonius Hospital between March 19, 2020 and May 5, 2020. Diagnosis of COVID-19 was made on the basis of a positive SARS-CoV-2 PCR except for three cases with clinical characteristics and a high-resolution computed tomography (HRCT) of the chest congruent with COVID-19 pneumonia. We collected demographics, clinical characteristics, radiology and survival data from medical hospital records. Severe disease was arbitrarily defined by hospitalization with the need for oxygen supplementation.

The control group consisted of 611 Dutch white healthy controls, from the biobank. The study was approved by The Medical research Ethics Committees United (MEC-U) of St. Antonius Hospital and all patients provided written informed consent (approval number R05-08A).

For validation we obtained data from the UK biobank (13). The validation cohort consisted of unrelated UK Biobank participants (application 44046) of European ancestry with 436 adult patients with a diagnosis of COVID-19 based on a positive SARS-CoV-2 PCR in the period 16 March−14 April 2020. In this period, testing was almost exclusively restricted to patients admitted to the hospital or presenting at emergency services with severe disease plus healthcare workers suffering clinical signs of infection, including fever and cough or shortness of breath. Overall, for the UK, the case fatality rate was highest during the study period (https://ourworldindata.org/mortality-risk-covid?country~GBR). Death due to COVID-19 was calculated using ICD-10 codes U071 and U072 before end of May 2020 and 117 out of 436 (27%) of the UK biobank case cohort died due to COVID-19. This indicates that the test criteria at that time were a reasonable proxy for severe COVID-19. Furthermore, 356,799 UK biobank controls were included. All UK Biobank participants provided written informed consent, the UK Biobank study was approved by the National Research Ethics Service Committee North West-Haydock (REC reference 16/NW/0274), and all study procedures were performed in accordance with the World Medical Association Declaration of Helsinki ethical principles for medical research.

For replication we obtained summary data from the severe COVID-19 GWAS group (14) for white subjects. Replication cohort I consisted of 835 adult patients, of which 30% were female and a median age of 65 (IQR 56–75) years hospitalized with COVID-19 in Italy and 1,255 controls of which 39% is female and a median age of 49 (IQR 33–59) years. Replication cohort II consisted of 775 adult patients, of which 34% were female and a median age of 67 (IQR 58–75) years hospitalized with COVID-19 in Spain and 950 controls of which 33% were female and a median age of 44 (IQR 33–50) years. Severe COVID-19 was defined as hospitalization with respiratory failure. Information about respiratory support and comorbidities were described previously in more detail (14).

### Genotyping

For Dutch subjects, DNA was extracted using a Chemagic 360 (PerkinElmer, Waltham, Massachusetts, USA) from whole blood. The discovery cohort was genotyped for *MUC5B* rs35705950 genotype with a pre-designed taqman SNP genotyping assay and the QuantStudio® 5 Real-Time PCR system (both ThermoFisher Scientific, Waltham, Massachusetts, USA).

For the UK biobank data, we obtained genotype counts summarized separately for cases and controls for SNP rs35705950, with data from participants who died before the epidemic excluded. SNP data was generated from the Affymetrix Axiom UK Biobank array and the UK BiLEVE array following extensive central quality control (13). We used genetic data from the “v3” release of UKBB containing the full set of Haplotype Reference Consortium (HRC) and 1000 Genomes imputed variants, followed by additional internal quality control to define a cohort of unrelated white European participants (15).

For replication cohort I and II, we obtained genotype counts summarized separately for white cases and controls from the severe COVID-19 GWAS group for SNP rs35705950 at chr11, pos_hg38 1219991, G, T (www.c19-genetics.eu). SNP rs35705950 was directly genotyped, except for 3 out of 2,090 genotypes of the Italian cohort. These were imputed via TOPMed reference panel (14).

### Statistical Analysis

SPSS 24 (IBM, Armonk, New York, USA) was used for statistical analysis. Due to ethnic differences in the prevalence of the *MUC5B* rs35705950 alleles, genetic analyses were stratified by ethnicity and only statistically analyzed in white subjects. Differences between white and non-white patients and between carriers and non-carriers of the rs35705950 T-allele were calculated using a Chi square test for categorical data. Differences with continuous data were tested with *t*-test or the Mann-Whitney U test where appropriate. Differences between the allele and genotype frequencies were calculated with the Pearson's goodness-of-fit chi-square test, together with the OR and 95% CI. Binary logistic regression was used to test for *MUC5B* rs35705950 association and COVID-19 with age and sex as confounding variables. Linear regression was used to test for rs35705950 association with age, adjusted for sex. Fisher's exact test was used to test for deviation from Hardy–Weinberg equilibrium. A value of *p* < 0.05 was considered statistically significant. Meta-analyses were performed using the allele contrast and dominant model in the web tool META-Genyo (16). Heterogeneity in the data was evaluated with *I*^2^ statistics and Cochran's Q test was low for both the allele contrast and dominant model. The fixed-effect estimate method, inverse variance was used.

## Results

### Dutch Participants

In total 108 patients hospitalized with COVID-19 (Table 1) in The Netherlands were included in the study of which 74 (69%) were males. Among 108 patients, 83 (77%) were white and 25 patients were non-whites. The median age of the patients was 66 years (range 19.1–92.4) and differed significantly between whites (71 years) and non-whites (55 years; *p* = 0.0004). Of all patients, 24 patients were admitted to the intensive care unit (22%).The median length of hospitalization of patients who survived COVID-19 was 9 days. Twenty-three patients died (21%) and they were significantly older than patients who survived, 74 vs. 63 years, respectively (*p* = 0.002). There was a trend toward significance for a younger age at death in non-whites (Table 1).

**Table 1:** Characteristics of Dutch patients hospitalized with COVID-19.

	**All**	**White**	**Non-white**	***p***
*N*	108	83	25	
Males, *n* (%)	74 (69)	56 (67)	18 (72)	0.67
Age at diagnosis, median (IQR), y	66 (22)	71 (18)	55 (14)	0.001
Stay at ICU, *n* (%), days	24 (22)	18 (22)	6 (24)	0.79
Deaths, *n* (%)	23 (21)	18 (14)	5 (20)	0.86
Age at death, median (IQR), y	75 (15)	76 (14)	64 (24)	0.26
Length of hospitalization survivors, median (IQR), days	9 (10)	11 (14)	7.5 (5)	0.10
Body Mass Index, median (IQR)	28.1 (5)	28.1 (4.6)	28.2 (7.2)	0.72
Diabetes, *n* (%)	8 (7)	7 (8.4)	1 (4)	0.68
Asthma/COPD, *n* (%)	16 (15)	15	1	0.11
Interstitial lung disease, *n* (%)	1 (1)	1 (1)	0(1)	1.00
Pulmonary hypertension, *n* (%)	1 (1)	1 (1)	0 (1)	1.00

*N, number; y, years; IQR, inter quartile range; ICU, intensive care unit; COPD, chronic obstructive pulmonary disease*.

The control cohort consisted of 611 white subjects with a median age of 59 years, of which 285 (47%) were male.

### UK Biobank Participants

In total 436 patients of European ancestry with a diagnosis of COVID-19 were included in the study of which 249 (57%) were males. Characteristics and co-morbidities for UK biobank participants are presented in Table 2. The median age of the patients was 70 years. One hundred and seventeen patients (27%) patients died. The control cohort consisted of 356,799 subjects with a median age of 69 years, of which 161,178 (45%) were male. Significantly more male sex, older age, higher number of death, higher BMI, more diabetes, COPD and ILD were observed among COVID-19 cases when compared with controls (Table 2).

**Table 2:** Characteristics of UK biobank participants.

	**COVID-19**	**Controls**	***p* ^$^**
*N*	436	356,799	
Males, *n* (%)	249 (57)	161,179 (45)	<0.001
Age, median (IQR), y^∧^	70 (16)	69 (13)	<0.001
Deaths, *n* (%)^*^	117 (27)	688 (0.002)	<0.001
Age at death, median (IQR), y	75 (8)	74(8)	0.75
Body Mass Index, median (IQR)	29.1 (6.2)	27.3 (5.7)	<0.001
Diabetes, *n* (%)	38 (8.7)	15,815 (4.4)	<0.001
Asthma, *n* (%)	65 (15)	41,508 (12)	0.015
COPD, *n* (%)	42 (9.6)	8,371 (2.4)	<0.001
Interstitial lung disease, *n* (%)	7 (1.6)	1,262 (0.3)	<0.001

*N, number; y, years; IQR, inter quartile range; COPD, chronic obstructive pulmonary disease; Diabetes, any definition of diabetes*.

$ *Linear regression associations observed using available data in white European covid cases vs. controls, adjusted for age and sex where relevant*.

∧ *=age at 15/03/2020*

* *Deaths were those that occurred between 15/03/20 and 31/5/20 only*.

### Association of *MUC5B* Rs35705950 With COVID-19

In the discovery cohort of 108 patients, there were 99 patients with a GG genotype and 9 patients with a GT genotype. The minor T-allele frequency of the *MUC5B* promoter polymorphism was 0.04. In the white subgroup of COVID-19 patients, 76 had a GG genotype and 7 a GT genotype, which was in Hardy-Weinberg equilibrium. The frequency of the T-allele in the white COVID-19 group was 0.04 and this was significantly lower than the T-allele frequency of 0.10 in the control group (*p* = 0.023; OR = 0.42, CI = 0.19–0.91; Table 3). Age and sex were significantly different between cases and controls, however multivariate analysis with age and sex as confounding variables showed comparable results (*p* = 0.03; OR = 0.40, CI = 0.18–0.91).

**Table 3:** *MUC5B* rs35705950 genotype in white subjects with severe COVID-19 and controls.

	**Discovery**		**Validation**		**Replication I**		**Replication II**	
	**COVID-19**	**Controls**	**COVID-19**	**Controls**	**COVID-19**	**Controls**	**COVID-19**	**Controls**
Country	Netherlands	Netherlands	United Kingdom	United Kingdom	Italy	Italy	Spain	Spain
*N*	83	611	436	356,735	835	1,255	775	950
GG, *n* (%)	76 (92)	501 (82)	369 (85)	281,333 (79)	670 (80)	964 (77)	624 (81)	723 (76)
GT, *n* (%)	7 (8)	103 (17)	67 (15)	70,987 (20)	156 (19)	268 (21)	140 (18)	210 (22)
TT, *n* (%)	0 (0)	7 (1)	0 (0)	4,415 (8)	9 (1)	23 (2)	11 (1)	17 (2)
MAF	0.04	0.10	0.08	0.11	0.10	0.13	0.10	0.13
OR	0.42		0.66		0.81		0.79	
95% CI	0.19–0.91		0.51–0.85		0.67–0.99		0.64–0.98	
*p*	0.023		0.001		0.039		0.030	

*N, number; MAF, minor allele frequency; OR, odds ratio; CI, confidence interval*.

For the UK validation cohort the minor T-allele frequency of *MUC5B* rs35705950 was 0.08 in cases and this was significantly lower than the T-allele frequency of 0.11 in the controls (*p* = 0.001; OR = 0.66, CI = 0.51–0.85; Table 3).

This association remained significant after adjustment for age, sex, BMI, asthma, COPD, ILD and diabetes (Table 4).

**Table 4:** Multivariate association analysis for COVID-19 in the UK biobank validation cohort.

	**Odds ratio**	**95% CI**	***p***
rs35705950 T allele	0.67	0.52–0.86	0.002
Sex	1.52	1.26–1.84	<0.001
Age	1.02	1.01–1.03	0.002
Body Mass Index	1.06	1.04–1.08	<0.001
Diabetes	1.22	0.85–1.74	0.28
COPD	3.20	2.26–4.52	<0.001
Asthma	1.11	0.84–1.46	0.47
Interstitial lung disease	2.45	1.08–5.58	0.03

*CI, confidence interval; COPD, chronic obstructive pulmonary disease*.

For the UK biobank cohort we separately investigated if the rs35705950 allele frequency associated with age. The association of the rs35705950 allele frequency with age is small with β = −0.0002 (*p* = 0.027) with a small decrease in T-allele frequency with increasing age. Moreover, if we remove ILD cases, the association for non-ILD UK biobank participants become β = −0.0003 (*p* = 2.8 × 10^−4^). This indicates that there is no survival bias of T-allele carriers. Our data also demonstrates no survival bias for ILD cases during this period of isolation.

In the severe COVID-19 replication cohort I and cohort II, the minor T-allele frequency of *MUC5B* rs35705950 was 0.10 and this was significantly lower than the T-allele frequency of 0.13 in both control cohorts (cohort I: *p* = 0.039; OR = 0.81, CI = 0.67–0.99; and cohort II: *p* = 0.030; OR = 0.79, CI = 0.64–0.98; Table 3). Analysis of replication cohort I and II together, adjusted for sex, age and top 10 principal component showed comparable results, OR 0.75 (SD 0.098); *p* = 0.003.

**Figure 1:**
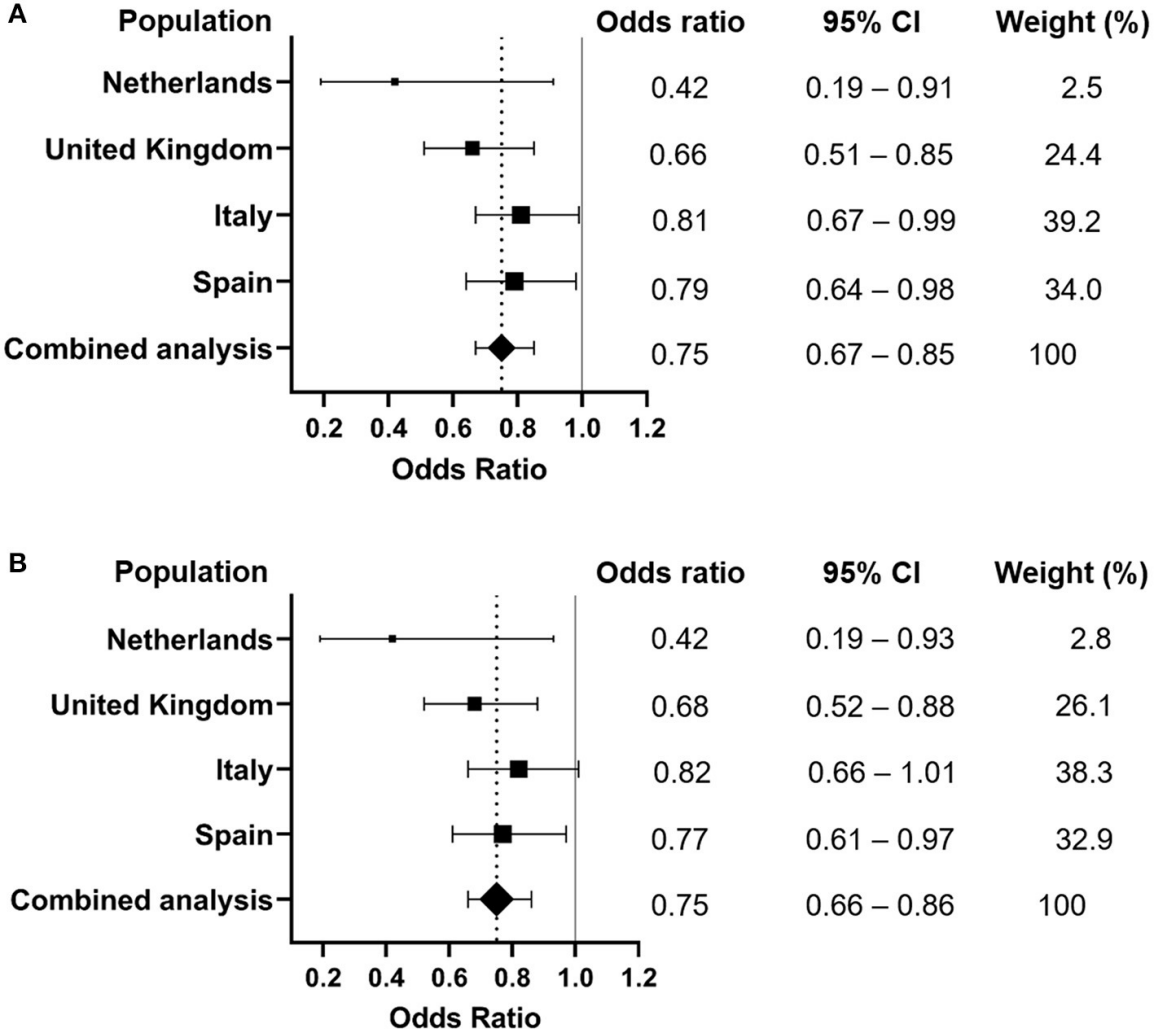
Forest plots of meta-analyses of the association of *MUC5B* rs35705950 with severe COVID-19 in subjects of European ancestry. Dotted line represents the odds ratio from the combined analysis. **(A)** Meta-analysis of allele contrast (T vs. G; P combined analysis = 6.63 e-06). **(B)** Meta-analysis of the dominant T-allele carriage model (GT+TT vs. GG; P combined analysis = 2.05e-05). CI, confidence interval.

Meta-analyses were performed to analyze the association of *MUC5B* rs35705950 with severe COVID-19, both for comparison of allele contrast and for a dominant T-allele carriage model (GT+TT vs. GG). Figure 1A shows the forest plot of the T vs. G allele meta-analysis of the four cohorts. The pooled negative association with COVID-19 for the T allele was significant with an OR of 0.75 (CI: 0.67–0.85); *p* = 6.63 × 10^−6^. Figure 1B shows the forest plot of the meta-analysis of the dominant model (GT+TT vs. GG) of the four cohorts. The pooled negative association of T allele carriage was significant with an OR of 0.75 (CI: 0.66-0.86; *p* = 2.05 × 10^−5^).

## Discussion

In this study we used a candidate gene case-control approach to examine whether a genetic polymorphism that influences expression of MUC5B is associated with susceptibility to severe COVID-19. We observed a significant association between the *MUC5B* rs35705950 promoter polymorphism and severe COVID-19 in four white European cohorts. The results of the meta-analyses demonstrate the protective effect of the *MUC5B* T allele against severe COVID-19. The T-allele frequency and T-carrier frequency was lower in severe COVID-19 patients than in controls.

Beneficial effects of carriage of the T-allele have been reported before. In smoking non-Hispanic white COPD patients with interstitial HRCT features, T-allele carriers experienced less acute respiratory disease and a longer time-to-first event (17). Furthermore, in IPF patients, carriers had a lower bacterial burden than non-carriers (18) and better survival (19).

In the human respiratory system, MUC5B is secreted throughout the lung by submucosal glands and the superficial epithelium of trachea, bronchi, bronchioles and alveoli, and by salivary glands and nasal mucosa (3, 4, 20, 21). The T-allele was shown to increase the MUC5B promoter activity and carriers of the T-allele demonstrated increased RNA expression of MUC5B in lung tissue (7, 8, 22). A recent paper shows that MUC5B rs35705950 resides within a gene enhancer that is subject to epigenetic remodeling (23). In the airway epithelium of an explant lung of a severe ICU admitted COVID-19 patient, dramatically reduced MUC5B protein and mRNA expression was found when compared with control lung (24).

The increased MUC5B production in T-allele carriers may protect carriers from adverse events related to airway defense. This may be of particular importance in aging, because mucus production and mucociliary clearance have been described to decrease with aging (5, 6). Furthermore, decreased mucociliary clearance may underlie the observed age-related increase in the incidence of severe community-acquired pneumonia in the elderly (25). Similar to previous reports on COVID-19 (1) we also observed that severe COVID-19 and death from COVID-19 is predominantly found in the elderly.

Severe COVID-19 is primarily associated with a respiratory system pathology. Autopsy of patients who died from COVID-19 showed presence of diffuse chronic and tracheobronchial inflammation and alveolar type 2 (AT2) cell hyperplasia in the majority of biopsies (26). SARS-CoV-2 virus was detected in both airway epithelium and AT2 cells and the authors concluded that histology suggests progressive disease that begins in the airway and extends to alveolar zones (26). High constitutive levels of MUC5B in the elderly may protect the airway from SARS-CoV-2 viral infection.

Death rates of the Dutch and UK COVID-19 cases are high. The UK biobank positive COVID-19 cases even had a death rate of 27% which is considerably higher than the maximum 15.2% (27) evolving case fatality rate in the UK from mid-March to end May 2020. After community testing was rolled out, the case fatality rate in the UK decreased below 4% (27). The high death rate of affected UK biobank cases may be due to the older age of biobank participants with a median of 69 years old. With increasing age, the lung changes to the extent that alleles which in younger people confer non-essential divergent expression, may influence the risk of disease in aged tissue. In aging lung diseases such as IPF, COPD and lung cancer, a pattern is emerging of shared disease loci. Although loci are shared, it is of considerable interest that the respective diseases associate with opposite risk alleles and with opposing expression levels (10, 11, 28). Previously we summarized findings and presented a theory in which trade-offs in the aging respiratory system exist (11). The present study shows that the *MUC5B* rs35705950 polymorphism may be added to this list of shared loci with opposite risk alleles. The *MUC5B* T-allele, which appears beneficial in this study, is best known as a major risk allele for IPF (7). IPF is a rare non-infectious pulmonary aging disease of unknown cause characterized by insidious onset of disease in patients without a history of pulmonary health problems. Subsequent studies showed that the *MUC5B* T-allele not only predisposes to IPF but to a variety of chronic progressive forms of pulmonary fibrosis (29–32).

Because severe COVID-19 is associated with substantial pneumonitis and shares multiple risk factors with IPF, Fadista et al. recently investigated whether a genetic correlation between IPF and severe COVID-19 exists using a Mendelian randomization approach (33). They found that genetically increased risk of IPF indeed associated with increased COVID-19 severity, except for the *MUC5B* allele. The *MUC5B* risk allele had a different effect compared with other IPF predisposing alleles and protected against COVID-19 hospitalization in the elderly. Because the *MUC5B* results contradicted their hypotheses the authors were concerned that the analysis might have been influenced by possible selection bias: 1) due to the rs35705950 T allele carriers undertaking strict self-isolation, and 2) due to survival bias of the rs35705950 non-IPF T allele carriers (33). With the unique data of the UK biobank cohort, we were able to address these questions. First, the *MUC5B* T allele is only known to be associated with progressive fibrosing ILD. These patients may have been isolating due to clinical vulnerability. However, instead of underrepresentation, we were able to show the significant overrepresentation of patients with ILD in the biobank COVID-19 cases vs. non-cases. These data include 5 IPF cases and 1,014 IPF controls, bias introduced by effective shielding of these patients is therefore not present. Second, it is suggested that *MUC5B* T-allele carriers may have increased survival in the population. This would result in increasing T-allele frequencies with age. However, we found a very slight decrease in the T-allele frequency with age. When we delete ILD subjects from the cohort, the association remained, meaning that there is no survival bias of T-allele carriers in the non-ILD population.

The finding by Fadista et al. is in line with our current findings and we found no evidence for stratification bias driving these results. We performed a candidate allele study because we hypothesized that the IPF predisposing allele would protect against COVID-19 and confirmed this hypothesis. The current finding of protection against severe COVID-19 combined with the established increased risk for pulmonary fibrosis in T-allele carriers may represent a trade-off that becomes apparent with aging. During the first decades of life the effect of both alleles may be neutral while at an older age differences in constitutive expression levels may predispose to disease. The pleiotropic property of the gene polymorphism is expressed only in the older individuals. This idea complements the well-established principle of pleiotropic antagonism, the theory of aging where one gene is involved in multiple traits (pleiotropy) with a beneficial fitness enhancing effect in early life and a detrimental fitness diminishing effect in later life (34).

A limitation of the study is the focus on white European populations. Minor allele frequencies for *MUC5B* rs35705950 are known to differ between populations. The allele frequencies of the control cohorts are congruent with previous reports (32, 35–37). The protective T-allele is known to have the highest frequency in populations of European ancestry, but is less frequent to non-existent in non-European populations. It is tempting to speculate that the increased risk for infection with SARS-CoV-2 and the worse clinical outcome in black, Asian and minority ethnic populations in western societies (38) may be associated with low carriage of the protective *MUC5B* T-allele. Replication cohort I and II are part of the study population used by Fadista et al. (33). In their study, a 89% white patient population and a 99% white control cohort was used, therefor they adjusted the analysis for genetic ancestry principal components (33). We used a white Dutch and UK population, so in order to replicate our findings and allow comparison of the results we included replication cohorts I and II and performed the analysis on white subjects only, which produced similar results. However, future studies aimed at improving understanding of COVID-19 risk in populations worldwide should include genetics of different ethnic groups. Another limitation is the small sample size of the Dutch cohort, yielding a significant result but with a wide confidence interval. Furthermore, specific information on disease severity such as organ involvement, CO-RAD and CT-severity scores are missing. However, all patients in the Dutch cohort were hospitalized for confirmed COVID-19 and had a SpO_2_ <94%. Hospital triage during the study period was restricted because the wards were overcrowded. Furthermore, 22% of patients were admitted to the intensive care unit and 24% of patients died. In addition, the death rates of the UK COVID-19 cases was high (27%), all suggestive of case cohorts with severe COVID-19. However, further studies are needed to investigate if the *MUC5B* polymorphism will associate with specific COVID-19 severity scores.

A strength of our study is the inclusion of the UK Biobank cohort, with cases and controls having been recruited as one cohort 9–13 years prior to the COVID pandemic. This procedure is most ideal to avoid recruitment bias in case-control studies and the cohort yields a highly significant result.

In conclusion, we found that carriage of the T-allele of *MUC5B* rs35705950 confers protection from development of severe COVID-19. Because the T-allele is a known risk allele for pulmonary fibrosis, this study provides further evidence for the existence of trade-offs between optimal expression levels in the aging lung.

## Data Availability Statement

The datasets presented in this article are not readily available because they contain potentially identifying or sensitive patient information. Requests to access the datasets can be directed to the corresponding author.

## Ethics Statement

The ILD biobank and data registry of the St. Antonius Hospital was reviewed and approved by the Medical research Ethics Committees United (MEC-U) of St. Antonius Hospital. The UK Biobank study was reviewed and approved by the National Research Ethics Service Committee North West-Haydock. All participants provided their written informed consent to participate in this study.

## Author Contributions

The study was conceived and designed by CM and JV. CB, CS, AD, DE, and MQ contributed to the conduct of this study. Data were acquired by MQ, JV, AD, and DE and analyzed by AD and JV. CM reviewed the medical literature, oversaw the conduct of the study, participated in the interpretation of data, drafted, and wrote the manuscript. All authors reviewed and contributed to the manuscript during its development and approved it for publication.

## Funding

This study was funded by ZonMW TopZorg St Antonius Care grant nr 842002001; ZonMW Topspecialistische Zorg en Onderzoek grant no 10070012010004; Nederlandse Vereniging van Artsen voor Longziekten en Tuberculose COVID-19 grant; GW4 BioMed Medical Research Council Doctoral Training Partnership. Funders were not involved in the study design, collection, analysis and interpretation of the data.

## Conflict of Interest

The authors declare that the research was conducted in the absence of any commercial or financial relationships that could be construed as a potential conflict of interest.

## Publisher's Note

All claims expressed in this article are solely those of the authors and do not necessarily represent those of their affiliated organizations, or those of the publisher, the editors and the reviewers. Any product that may be evaluated in this article, or claim that may be made by its manufacturer, is not guaranteed or endorsed by the publisher.

## References

[1] Rosenberg ES Dufort EM Blog DS Hall EW Hoefer D Backenson BP. COVID-19 testing, epidemic features, hospital outcomes, and household prevalence, New York State-March 2020. Clin Infect Dis. (2020) 71:1953–9. 10.1093/cid/ciaa549 32382743 PMC7239264

[2] Knowles MR Boucher RC. Mucus clearance as a primary innate defense mechanism for mammalian airways. J Clin Invest. (2002) 109:571–7. 10.1172/JCI0215217 11877463 PMC150901

[3] Okuda K Chen G Subramani DB Wolf M Gilmore RC Kato T. Localization of secretory mucins MUC5AC and MUC5B in normal/healthy human airways. Am J Respir Crit Care Med. (2019) 199:715–27. 10.1164/rccm.201804-0734OC 30352166 PMC6423099

[4] Roy MG Livraghi-Butrico A Fletcher AA McElwee MM Evans SE Boerner RM. Muc5b is required for airway defence. Nature. (2014) 505:412–6. 10.1038/nature12807 24317696 PMC4001806

[5] Grubb BR Livraghi-Butrico A Rogers TD Yin W Button B Ostrowski LE. Reduced mucociliary clearance in old mice is associated with a decrease in muc5b mucin. Am J Physiol Lung Cell Mol Physiol. (2016) 310:L860–7. 10.1152/ajplung.00015.2016 26968767 PMC4867354

[6] Svartengren M Falk R Philipson K. Long-term clearance from small airways decreases with age. Eur Respir J. (2005) 26:609–15. 10.1183/09031936.05.00002105 16204590

[7] Seibold MA Wise AL Speer MC Steele MP Brown KK Loyd JE. A common MUC5B promoter polymorphism and pulmonary fibrosis. N Engl J Med. (2011) 364:1503–12. 10.1056/NEJMoa1013660 21506741 PMC3379886

[8] Nakano Y Yang I V. Walts AD Watson AM Helling BA Fletcher AA. MUC5B promoter variant rs35705950 affects MUC5B expression in the distal airways in idiopathic pulmonary fibrosis. Am J Respir Crit Care Med Am Thorac Soc. (2016) 193:464–6. 10.1164/rccm.201509-1872LE 26871673 PMC4803086

[9] Raghu G Remy-Jardin M Myers JL Richeldi L Ryerson CJ Lederer DJ. Diagnosis of idiopathic pulmonary fibrosis An Official ATS/ERS/JRS/ALAT Clinical practice guideline. Am J Respir Crit Care Med. (2018) 198:e44–68. 10.1164/rccm.201807-1255ST 30168753

[10] Hobbs BD de Jong K Lamontagne M Bossé Y Shrine N Artigas MS. Genetic loci associated with chronic obstructive pulmonary disease overlap with loci for lung function and pulmonary fibrosis. Nat Genet. (2017) 49:426–32. 10.1038/ng.3752 28166215 PMC5381275

[11] Van Moorsel CHM. Trade-offs in aging lung diseases: a review on shared but opposite genetic risk variants in idiopathic pulmonary fibrosis, lung cancer and chronic obstructive pulmonary disease. Curr Opin Pulmonary Med. (2018) 24:309–17. 10.1097/MCP.0000000000000476 29517586 PMC5895171

[12] Gluckman PD Low FM Buklijas T Hanson MA Beedle AS. How evolutionary principles improve the understanding of human health and disease. Evol Appl. (2011) 4:249–63. 10.1111/j.1752-4571.2010.00164.x 25567971 PMC3352556

[13] Bycroft C Freeman C Petkova D Band G Elliott LT Sharp K. The UK Biobank resource with deep phenotyping and genomic data. Nature. (2018) 562:203–9. 10.1038/s41586-018-0579-z 30305743 PMC6786975

[14] Ellinghaus D Degenhardt F Bujanda L Buti M Albillos A Invernizzi P. Genomewide association study of severe covid-19 with respiratory failure. N Engl J Med. (2020) 383:1522–34. 10.1056/NEJMoa2020283 32558485 PMC7315890

[15] Tyrrell J Mulugeta A Wood AR Zhou A Beaumont RN Tuke MA. Using genetics to understand the causal influence of higher BMI on depression. Int J Epidemiol. (2019) 48:834–48. 10.1093/ije/dyy223 30423117 PMC6659462

[16] Martorell-Marugan J Toro-Dominguez D Alarcon-Riquelme ME Carmona-Saez P. MetaGenyo: a web tool for meta-analysis of genetic association studies. BMC Bioinformatics. (2017) 18:563. 10.1186/s12859-017-1990-4 29246109 PMC5732412

[17] Ash SY Harmouche R Putman RK Ross JC Martinez FJ Choi AM. Association between acute respiratory disease events and the MUC5B promoter polymorphism in smokers. Thorax. (2018) 73:1071–4. 10.1136/thoraxjnl-2017-211208 29440587 PMC6089672

[18] Molyneaux PL Cox MJ Willis-Owen SAG Mallia P Russell KE Russell AM. The role of bacteria in the pathogenesis and progression of idiopathic pulmonary fibrosis. Am J Respir Crit Care Med. (2014) 190:906–13. 10.1164/rccm.201403-0541OC 25184687 PMC4299577

[19] Peljto AL Zhang Y Fingerlin TE Shwu-Fan M Garcia JGN Richards TJ. Association between the MUC5B promoter polymorphism and survival in patients with idiopathic pulmonary fibrosis. JAMA. (2013) 309:2232–9. 10.1001/jama.2013.5827 23695349 PMC4545271

[20] Nielsen PA Bennett EP Wandall HH Therkildsen MH Hannibal J Clausen H. Identification of a major human high molecular weight salivary mucin (MG1) as tracheobronchial mucin MUC5B. Glycobiology. (1997) 7:413–9. 10.1093/glycob/7.3.413 9147051

[21] Hancock LA Hennessy CE Solomon GM Dobrinskikh E Estrella A Hara N Evans CM. Muc5b overexpression causes mucociliary dysfunction and enhances lung fibrosis in mice. Nat Commun. (2018) 9:5363. 10.1038/s41467-018-07768-9 30560893 PMC6299094

[22] Helling BA Gerber AN Kadiyala V Sasse SK Pedersen BS Sparks L Schwartz DA. Regulation of MUC5B expression in idiopathic pulmonary fibrosis. Am J Respir Cell Mol Biol. (2017) 57:91–9. 10.1165/rcmb.2017-0046OC 28272906 PMC5516283

[23] Gally F Sasse SK Kurche JS Gruca MA Cardwell JH Okamoto T Schwartz DA Gerber AN. The MUC5B-associated variant rs35705950 resides within an enhancer subject to lineage- And disease-dependent epigenetic remodeling. JCI Insight. (2021) 6:e144294. 10.1172/jci.insight.144294 33320836 PMC7934873

[24] Yin W Cao W Zhou G Wang L Sun J Zhu A. Analysis of pathological changes in the epithelium in COVID-19 patient airways. ERJ Open Res. (2021) 7:00690-2020. 10.1183/23120541.00690-2020 33829055 PMC7898030

[25] Fung HB Monteagudo-Chu MO. Community-acquired pneumonia in the elderly. Am J Geriatr Pharmacother. (2010) 8:47–62. 10.1016/j.amjopharm.2010.01.003 20226392

[26] Borczuk AC Salvatore SP Seshan S V. Patel SS Bussel JB Mostyka M. COVID-19 pulmonary pathology: a multi-institutional autopsy cohort from Italy and New York City. Mod Pathol. (2020) 33:2156–68. 10.1038/s41379-020-00661-1 32879413 PMC7463226

[27] Roser M Ritchie H Ortiz-Ospina E Hasell J. Coronavirus Pandemic (COVID-19). OurWorldInData.org (2020). Available online at: https://ourworldindata.org/coronavirus

[28] Snetselaar R van Oosterhout MFM Grutters JC Van Moorsel CHM. TERT polymorphism rs2736100: a balancing act between cancer and non-cancer disease, a meta-analysis. Front Med. (2018) 5:41. 10.3389/fmed.2018.00041 29536006 PMC5835035

[29] Van Der Vis JJ Snetselaar R Kazemier KM Ten Klooster L Grutters JC Van Moorsel CHM. Effect of Muc5b promoter polymorphism on disease predisposition and survival in idiopathic interstitial pneumonias. Respirology. (2016) 21:712–7. 10.1111/resp.12728 26699835

[30] Ley B Newton CA Arnould I Elicker BM Henry TS Vittinghoff E. The MUC5B promoter polymorphism and telomere length in patients with chronic hypersensitivity pneumonitis: an observational cohort-control study. Lancet Respir Med. (2017) 5:639–47. 10.1016/S2213-2600(17)30216-3 28648751 PMC5555581

[31] Platenburg MGJP Wiertz IA van der Vis JJ Crestani B Borie R Dieude P. The MUC5B promoter risk allele for idiopathic pulmonary fibrosis predisposes to asbestosis. Eur Respir J. (2020) 55:1902361. 10.1183/13993003.02361-2019 31949121

[32] Juge PA Lee JS Ebstein E Furukawa H Dobrinskikh E Gazal S. MUC5B promoter variant and rheumatoid arthritis with interstitial lung disease. N Engl J Med. (2018) 379:2209–19. 10.1056/NEJMoa1801562 30345907 PMC6371965

[33] Fadista J Kraven LM Karjalainen J Andrews SJ Geller F Baillie JK Jenkins RG Feenstra B. Shared genetic etiology between idiopathic pulmonary fibrosis and COVID-19 severity. EBioMedicine. (2021) 65:103277. 10.1016/j.ebiom.2021.103277 33714028 PMC7946355

[34] Austad SN Hoffman JM. Is antagonistic pleiotropy ubiquitous in aging biology? Evol Med Public Heal. (2018) 2018:287–94. 10.1093/emph/eoy033 30524730 PMC6276058

[35] Karczewski KJ Francioli LC Tiao G Cummings BB Alföldi J Wang Q. The mutational constraint spectrum quantified from variation in 141,456 humans. Nature. (2020) 581:434–43. 10.1530/ey.17.14.3 32461654 PMC7334197

[36] Borie R Crestani B Dieude P Nunes H Allanore Y Kannengiesser C. The MUC5B variant is associated with idiopathic pulmonary fibrosis but not with systemic sclerosis interstitial lung disease in the european Caucasian population. PLoS ONE. (2013) 8:e70621. 10.1371/journal.pone.0070621 23940607 PMC3734256

[37] López-Mejías R Remuzgo-Martínez S Genre F Pulito-Cueto V Rozas SMF Llorca J. Influence of MUC5B gene on antisynthetase syndrome. Sci Rep. (2020) 10:1415. 10.1038/s41598-020-58400-0 31996780 PMC6989632

[38] Pan D Sze S Minhas JS Bangash MN Pareek N Divall P. The impact of ethnicity on clinical outcomes in COVID-19: a systematic review. EClinicalMedicine. (2020) 23:100404. 10.1016/j.eclinm.2020.100404 32632416 PMC7267805

